# The complete mitochondrial genome of a stonefly species, *Suwallia bimaculata* (Plecoptera: Chloroperlidae)

**DOI:** 10.1080/23802359.2019.1660263

**Published:** 2019-09-02

**Authors:** Jinjun Cao, Ying Wang, Mengdan Chen, Mingyang Yuan, Weihai Li

**Affiliations:** Department of Plant Protection, Henan Institute of Science and Technology, Xinxiang, China

**Keywords:** *Suwallia bimaculata*, mitochondrial genome, phylogenetics

## Abstract

The stonefly *Suwallia bimaculata* belongs to the Chloroperlidae. The mitogenome of *S. bimaculata* was sequenced and annotated, the new representative of the complete mitogenome of the genus *Suwallia*. The entire genome of *S. bimaculata* is 16,125 bp totally with an A + T content of 68.5%, consisting of 13 protein-coding genes, two ribosomal RNAs, 22 transfer RNAs, and a 1,210 bp control region. All genes have the similar locations and strands with that of other published species of Plecoptera. Most PCGs use typical start/stop codon, whereas *ND5* use GTG as start codon. And, *COII* and *ND5* stopped with incomplete terminaton signal T. Phylogenetic analysis was performed using the Bayesian (BI) method and Maximum-likelihood (ML) methods based on 13 protein-coding genes and two ribosomal RNAs showed that *S. bimaculata* was the sister group to *Suwallia teleckojensis* and the clade *Suwallia* was closely to four perlodid species. More chloroperlid data was needed for further study of phylogeny in Chloroperlidae.

Most species of the genus *Suwallia* were treated by Alexander and Stewart (1999). The genus is now including 26 species and distributed in North America, Japan, Russia, Mongolia, and China (Li et al. 2015; Chen and Du 2018; Chen 2019; DeWalt et al. 2019). Prior to this study, the mitogenome of two species from Chloroperlidae have been sequenced and only one species *Suwallia teleckojensis* from China belongs to the genus *Suwallia* (Chen and Du 2015; Wang et al. 2018). Here, the mitochondrial genome of *Suwallia bimaculata*, which only distributed in Japan was sequenced and characterized by using next-generation sequencing method. Our study would facilitate future studies on the identification, population genetics, and phylogenetic analysis Chloroperlidae stoneflies.

The thorax muscle was extracted for the total genomic DNA using the QIAamp DNA Mini Kit (QIAGEN, Hilden, Germany). Total genome DNA was extracted from the adult specimen which was collected from Yamagata Prefecture (37.951°N, 139.698°E), Japan in July 2015 by Xingyue Liu. Voucher specimen’s genome DNA and the remaining stoneflies (No. Voh-0065) were deposited in the Institute of Entomology, Henan Institute of Science and Technology (HIST), Henan Province, China. We amplified and sequenced the complete mitogenome as described in previously studies (Wang et al. 2017; Liu et al. 2018; Cao et al. 2019), and the sequence reads were assembled into contigs using the BioEdit version 7.0.5.3 (Hall 1999).

The complete mitogenome of *S. bimaculata* 16,125 bp totally with an A + T content of 68.5%, which has been deposited into GenBank (the accession number is MN121757). Like other insect, the mitogenome of *S. bimaculata* encoded 37 genes, consisting of 13 protein-coding genes, two ribosomal RNAs, 22 transfer RNAs, and a 1210 bp control region. For the 37 genes, two ribosomal RNAs, four protein-coding genes (*ND1*, *ND4*, *ND4L*, and *ND5*) and eight transfer RNAs (Q, C, Y, F, H, P, L1, V) were located in J-strand, whereas the remaining protein-coding genes and transfer RNAs in N-strand. Most PCGs use the normal start/stop codon, whereas *ND5* use GTG as start codon and both *COII* and *ND5* terminated with incomplete signal T––. The A + T content of PCGs, tRNAs, rRNAs, and the control region was 66.4%, 69.6%, 72.0%, and 79.5%, respectively. The size of 22 transfer RNAs ranged from 65 to 71 bp, most of the transfer RNAs genes have cloverleaf secondary structure while the tRNAS1 gene lacks the dihydrouridine arm.

The phylogenetic topology tree of *S. bimaculata* performed based on the dataset from the sequences of 13 protein-coding genes and two ribosomal RNAs among 15 stoneflies (including one unpublished species *Etrocorema hochii*) and two species (*Cryptoperla stilifera* and *Styloperla spinicercia*) as outgroups. The Bayesian (BI) and maximum-likelihood (ML) methods conduct the same topology tree in Figure 1. The result showed that *S. bimaculata* was the sister group to *Suwallia teleckojensis* and the clade *Suwallia* represent Chloroperlidae was closely to other four Perlodidae species. In addition, more chloroperlid mitogenome data is needed for further to study the phylogenetic relationship.

**Figure 1.**
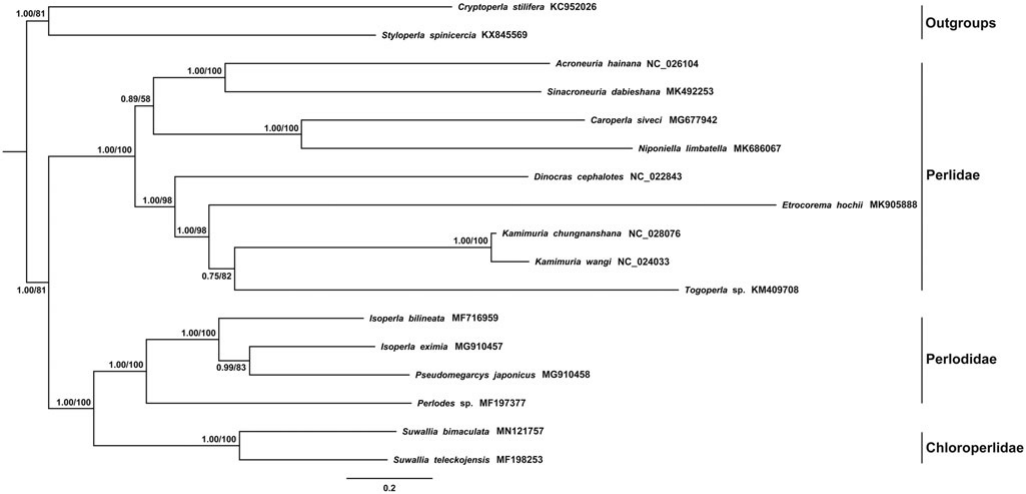
Phylogenetic analyses of *Suwallia bimaculata* based on the concatenated nucleotide sequences of the 13 protein-coding genes and two ribosomal RNAs among 17 stoneflies. The NCBI accession number for each species is indicated after the scientific name and numbers at nodes are bootstrap values.
